# Developing machine learning‐driven acute kidney injury predictive models using non‐standard EMRs in resource‐limited settings

**DOI:** 10.1002/mp.70038

**Published:** 2025-09-29

**Authors:** Shengwen Guo, Yuanhan Chen, Yu Kuang, Qin Zhang, Yanhua Wu, Zhen Xie, Ziqiang Chen, Qiang He, Feng Ding, Guohui Liu, Yuanjiang Liao, Chen Lu, Li Hao, Jing Sun, Lang Zhou, Rui Fang, Qingquan Luo, Haiquan Huang, Qi Cheng, Xinling Liang

**Affiliations:** ^1^ Department of Intelligent Science and Engineering School of Automation Science and Engineering South China University of Technology Guangzhou Guangdong China; ^2^ Department of Biomedical Engineering School of Material Science and Engineering South China University of Technology Guangzhou Guangdong China; ^3^ Department of Nephrology Guangdong Provincial People's Hospital (Guangdong Academy of Medical Sciences) Southern Medical University Guangzhou Guangdong China; ^4^ Department of Internal Medicine Second Ward, Nyingchi People's Hospital Tibet Autonomous Region China; ^5^ Medical Physics Program University of Nevada Las Vegas Nevada USA; ^6^ The Second School of Clinical Medicine Southern Medical University Guangzhou Guangdong China; ^7^ Department of Dermatology School of Medicine Sichuan Provincial People's Hospital University of Electronic Science and Technology of China Chengdu Sichuan China; ^8^ Department of Data Analysis Guangdong Easy Life Information Technology Co., Ltd Guangzhou Guangdong China; ^9^ Department of Nephrology the First Affiliated Hospital of Zhejiang Chinese Medical University (Zhejiang Provincial Hospital of Traditional Chinese Medicine) Hangzhou Zhejiang China; ^10^ Department of Nephrology Shanghai Ninth People's Hospital Shanghai Jiaotong University School of Medicine Shanghai China; ^11^ Department of Nephrology The Tenth Affiliated Hospital of Southern Medical University (Dongguan people's hospital) Dongguan Guangdong China; ^12^ Department of Nephrology Chongqing Ninth People's Hospital Chongqing China; ^13^ Department of Nephrology People's Hospital of Xinjiang Uygur Autonomous Region Urumqi Xinjiang China; ^14^ Department of Nephrology Second Hospital of Anhui Medical University Hefei Anhui China; ^15^ Department of Nephrology Second Hospital of Jilin University Changchun Jilin China; ^16^ Department of Electric Power Engineering School of Electric Power Engineering South China University of Technology Guangzhou Guangdong China

**Keywords:** acute kidney injury, early prediction, hospitalized patients, light gradient boosting machine, low‐ and middle‐income countries, non‐standard medical record

## Abstract

**Background:**

Acute Kidney Injury (AKI) remains a significant global health challenge, especially in resource‐limited settings. Most existing predictive models rely heavily on serum creatinine (SCr) levels and standardized electronic medical records (EMRs). However, in many low‐resource environments, SCr testing is infrequent, and EMR systems often lack standardization in data structure, terminology, and recording practices (a.k.a., non‐standard EMRs). These limitations hinder the consistent extraction of features needed for accurate AKI prediction and highlight the urgent need for adaptive frameworks tailored to diverse and resource‐limited healthcare environments.

**Purpose:**

This study aimed to develop and validate a machine learning model using non‐standardized EMRs for predicting AKI, even without SCr data.

**Methods:**

This multicenter observational study, conducted from 2010 to 2016 across 15 hospitals in China, employed the Light Gradient Boosting Machine (LightGBM) to create predictive models. The model's performance was assessed using area under the curve (AUC), precision, recall, specificity, and accuracy.

**Results:**

A total of 561 137 hospitalized patients were eligible for the analyses, of whom 45 610 were diagnosed with AKI. The LightGBM model demonstrated high accuracy in predicting AKI, with AUC values ranging from 0.860 to 0.986. The study showed that non‐standard EMRs could effectively predict AKI. Importantly, the model maintained strong predictive performance even without SCr data, indicating that AKI can be accurately predicted without this traditional biomarker.

**Conclusion:**

Non‐standard EMRs are valuable for predicting AKI, even in the absence of SCr data. This approach is particularly useful in resource‐limited settings, where traditional biomarkers are often unavailable, demonstrating the potential of other clinical features to compensate for missing SCr data in AKI prediction.

**List of Acronyms**
AKI: Acute kidney injury
ALB: Albumin
AP: Average precision
AUC: Using area under the curve
CCI: Charlson Comorbidity Index
Cl: Chloride
CO2CP: Carbon dioxide combining power
EMRs: Electronic medical records
GBDT: Gradient‐boosted decision tree
HCT: Hematocrit
HGB: Hemoglobin
K: Potassium
KDIGO: Kidney disease improving global outcomes
LightGBM: Light gradient boosting machine
LMICs: Low‐ and middle‐income countries
Na: Sodium
RASI: Renin angiotensin system inhibitors
SCr: Serum creatinine
SHAP: Shapley additive explanations
UA: Blood uric acid

## 1 INTRODUCTION

Acute kidney injury (AKI) is a global healthcare issue characterized by a sudden loss of kidney function, resulting in 1.7 million deaths annually, particularly in low‐ and middle‐income countries (LMICs) with limited healthcare resources.^1^ An estimated 21% of hospitalized patients worldwide develop AKI, which is associated with adverse outcomes, increased medical costs, and short‐ and long‐term morbidity and mortality.^2^, ^3^ In 2013, the International Society of Nephrology launched the Zero Preventable Deaths from Acute Kidney Injury by 2025 initiative (0by25).^4^ However, implementing this program in LMICs presents significant challenges due to limited healthcare infrastructure and insufficient resources for AKI diagnosis, management, and treatment.^4^

Timely identification of AKI in LMICs is crucial for stratifying high‐risk patients and facilitating appropriate clinical decisions for timely interventions, ultimately improving patient outcomes. Nevertheless, AKI is likely underdiagnosed in these regions. For instance, in Mainland China, AKI detection rates are reported as low as 10.7% using the 2012 Kidney Disease Improving Global Outcomes (KDIGO) criteria. This underestimation is partly due to infrequent serum creatinine (SCr) testing.^5^

SCr levels are pivotal for AKI diagnosis, necessitating repeated tests for early detection.^6^ In China, a representative LMIC, only 25.3% of hospitalized patients undergo at least two SCr tests, significantly lower than in developed countries (63.2%–67.6%).^7^ It is believed that this trend of low‐frequency detection of SCr also exists in other LMICs where an unexplained low prevalence of AKI has been reported.^5^, ^8^ This trend is not attributed to resource constraints, but rather to suboptimal local clinical practices and decision‐making processes.^5^, ^7^

Our previous survey among Chinese physicians highlighted a considerable gap in understanding renal diseases. Approximately half of the respondents were unfamiliar with AKI concepts and assessment methods.^9^ This lack of knowledge likely contributes to the infrequency of SCr testing in LMICs. Consequently, a reliable AKI predictive model is urgently needed, especially one that functions effectively with limited or no SCr data.

Machine learning methods have emerged as promising tools for early AKI detection by leveraging electronic health records and various clinical variables to predict kidney injury onset.^4^ These sophisticated algorithms can integrate data from diverse sources, including demographics, vital signs, and laboratory results, to generate more accurate and timely AKI predictions than traditional methods.^4^ Several models have demonstrated relatively high performance in estimating AKI risk among the general inpatient population.^10^, ^11^, ^12^ Notably, a DeepMind model employing a recurrent neural network achieved state‐of‐the‐art performance (area under the curve 0.92),^13^ while a gradient‐boosted decision tree (GBDT) model closely followed (area under the curve 0.89) in large academic hospital settings.^14^

Although DeepMind's and gradient‐boosted decision tree (GBDT)–based AKI detection algorithms have achieved impressive predictive accuracy in high‐resource settings, their reliance on frequent SCr measurements and standardized EMRs limits their applicability in low‐ and middle‐income countries (LMICs).^15^ By contrast, our work aims to fill this gap by developing an AKI prediction model that (1) operates on non‐standardized EMR data (i.e., heterogeneous, hospital‐specific systems with variable field names, units, and data‐entry workflows), and (2) does not require contemporaneous SCr values. This novel approach offers a practical decision‐support tool tailored to resource‐constrained environments.

In non‐standard EMRs, despite the lack of standard EMRs, several accessible records are still maintained in non‐standard EMR in resource‐limited settings, including laboratory tests (biochemical tests and complete blood counts), medications, and in‐hospital diagnoses. We hypothesized that the valuable information contained within these non‐standard EMRs could be utilized to develop a clinically translatable predictive model through machine learning for the early prediction of AKI. Consequently, this study aims to develop and validate a Light Gradient Boosting Machine (LightGBM)‐enabled diagnostic algorithm for the early prediction of AKI, using accessible EMRs without incorporating SCr level‐related features from a large cohort in hospitals at various levels in Mainland China. This approach provides a personalized immediate risk assessment tool for AKI that prompts tailored preventive action.

To the best of our knowledge, this is the first study to investigate an effective predictive model that excludes SCr level‐related features. To demonstrate the utility of the developed model, we compared its performance with and without the inclusion of SCr level‐related features. Such a diagnostic tool can be low‐cost, widely available, and clinically useful for predicting AKI onset within 24, 48, and 72 h for hospitalized patients, utilizing only over 100 features extracted from heterogeneous non‐standard EMR datasets. This approach has significant diagnostic and therapeutic implications from a precision medicine perspective.

Furthermore, we identified dominant features with strong discriminative ability for AKI prediction from non‐standard EMRs. We also demonstrated the utility of AKI models tailored exclusively to these most relevant features, extracted from non‐standard EMRs without incorporating SCr features. These models provided accurate AKI predictions despite limited data, even in the extreme condition of absence of repeated SCr examinations. This study offers valuable insights to facilitate the implementation of related clinical guidelines for early AKI prevention and treatment in clinics within LMICs.

## 2 METHODS

### 2.1 Data collection and extraction, transformation, and loading (ETL) process

#### 2.1.1 Data sources

**FIGURE 1:**
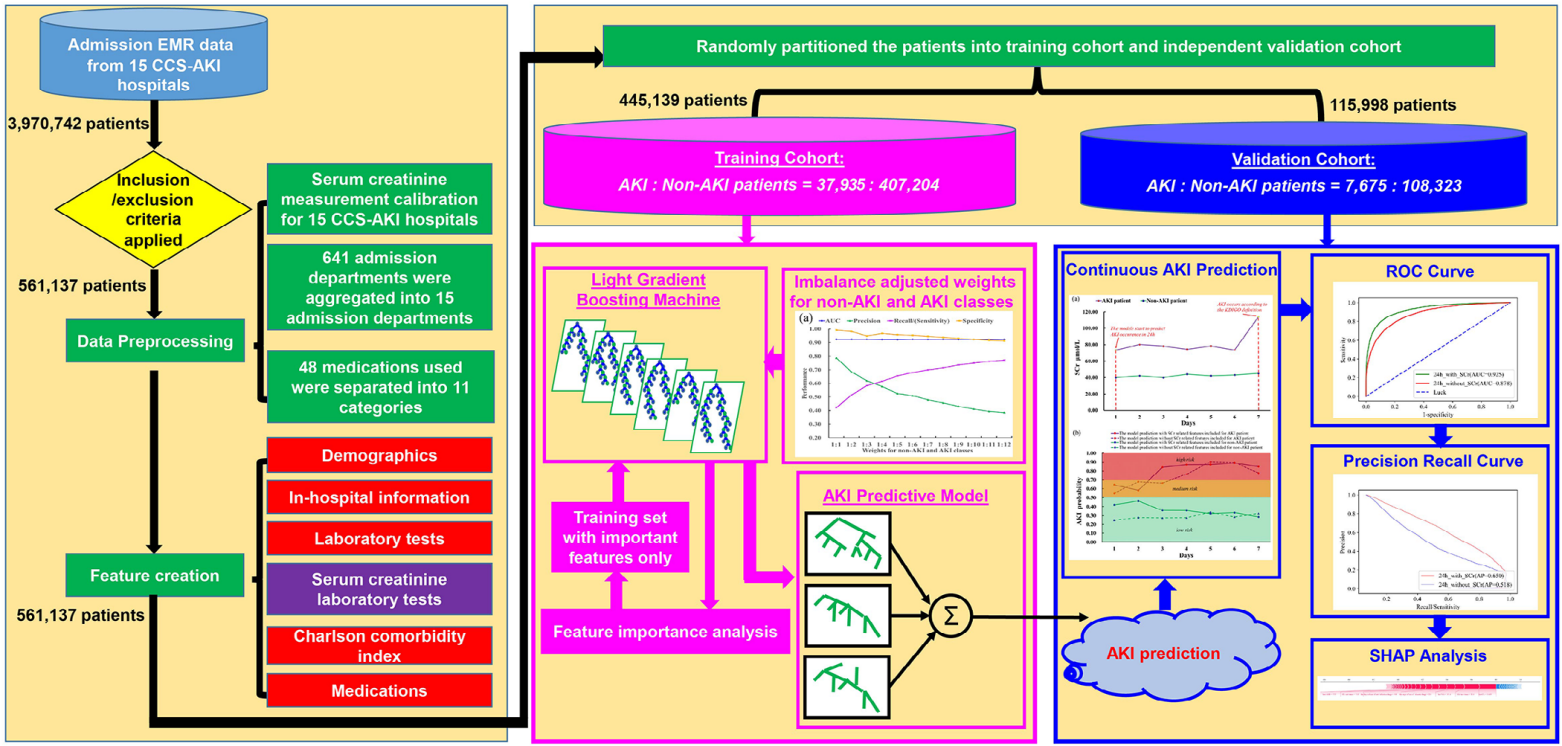
Study design for the development and validation of a LightGBM model to predict AKI. AKI, acute kidney injury; LightGBM, light gradient boosting machine; ROC, receiver operating characteristic; SHAP, Shapley additive explanations.

Figure 1 summarizes the study workflow and methods. All EMR data used in this study were derived from the China Collaborative Study on AKI (CCS‐AKI). This multicenter study recruited patients from 12 tertiary and 3 secondary hospitals in China between January 2010 and December 2016. To prepare the dataset for analysis, we applied the following exclusion criteria (exclusion criteria might overlap in some patients):

1. 11 951 admissions lacked any basic medical records;
2. 542 084 admissions were pediatric (< 18 years) or from pediatric wards;
3. 421 546 admissions had lengths of stay outside the 2–30 day window;
4. 2 462 491 admissions had fewer than two SCr measurements;
5. 67 546 admissions presented a baseline SCr > 353.6 µmol/L;
6. 690 390 admissions exhibited a maximum SCr < 53 µmol/L;
7. 85 540 admissions had Stage 5 chronic kidney disease. Stage 5 chronic kidney disease was defined as an eGFR < 15 mL/min/1.73 m^2^, calculated using the SCr‐based chronic kidney disease epidemiology Collaboration equation, and confirmed by a diagnosis of stage 5 chronic kidney disease, dialysis, or kidney transplantation;
8. 4036 admissions involved a history of limb amputation.

After applying these criteria, 561 137 adult admissions remained for analysis. These were split 80/20 into a training cohort (*n* = 445 139) and an independent test cohort (*n* = 115 998).

#### 2.1.2 Data extraction

From each hospital's EMR, we retrieved patient demographics, laboratory test results, medication records, Charlson Comorbidity Index (CCI), and timestamps of clinical events. All medical data were linked using a unique patient identifier, with personally identifiable information pre‐encrypted. Data extraction and cleaning were primarily conducted using structured query language (SQL) and Python.

#### 2.1.3 Transformation & standardization

1. Variable harmonization: Laboratory analyte names and measurement units were mapped to a unified glossary. Medication entries were consolidated into standardized drug classes (Table S1). Discharge diagnoses were coded uniformly using ICD‐10 criteria.^16^ All admission departments were grouped into 15 standardized admission departments, according to the Chinese Secondary Clinical Subject Standards in National Standard GBT 13745‐2009
  (https://www.chinesestandard.net/PDF/English.aspx/GBT13745‐2009).
2. SCr calibration: To correct inter‐laboratory variation in serum‐creatinine assays, we re‐measured 20–30 representative blood samples from each center at our reference laboratory. We then applied linear regression–based adjustment (per China Guidelines for the Comparability Verification of Quantitative Test Results in Medical Institutions, as well as the American Clinical and Laboratory Standards Institute's (CLSI) EP9‐A2 protocol)^17^, ^18^ to align all SCr values. If the correlation coefficient r is greater than or equal to 0.975, it is considered that the two hospitals have good test consistency, and no correction is needed. Otherwise, SCr values were then recalculated according to the fitting line.
3. Feature engineering: For each lab test, we computed summary statistics (first, last, minimum, maximum, mean, standard deviation, and test count). We also derived the Charlson Comorbidity Index (excluding moderate‐to‐severe renal disease weight) from discharge diagnoses (see Table S2). A total of 107 features were generated (see Table S3).

#### 2.1.4 Loading

The cleaned and standardized records were loaded into a secure, offline research database. This centralized dataset was accessible only to the study team for model training and evaluation; no live data entry occurred in this repository.

### 2.2 Division of cohorts

The patients were classified into the AKI group (*n* = 45 610) or non‐AKI group (*n* = 515 527). AKI patients were determined based on the KDIGO criteria^19^ (1) Increase in SCr by ⩾0.3 mg/dl (⩾26.5 µmol/L) within 48 h; or (2) increase in SCr to ⩾1.5 times baseline, which is known or presumed to have occurred within the prior 7 days. The SCr values from all the hospitals were calibrated by the Clinical Laboratory Center of Guangdong Provincial People's Hospital. All included patients’ serial SCr levels during the process of hospitalization were sorted in an ascending order according to the SCr test time points. The minimum SCr value was used for calculating eGFR as the baseline value to determine AKI based on the formula of Chronic Kidney Disease Epidemiology Collaboration.^20^

To account for geographic and institutional diversity, we randomly assigned data from 15 hospitals into training and independent test sets based on hospital level (See Cohort Division Strategy in Supporting Information). The training set comprised 11 hospitals (nine tertiary and two secondary), while the independent test set comprised the remaining four hospitals (three tertiary and one secondary). Both sets maintained the same AKI‐to‐non‐AKI ratio (1:11.3) as the overall cohort. Detailed hospital‐level sample sizes and AKI ratios are provided in Table S4.

### 2.3 Data collection windows

**FIGURE 2:**
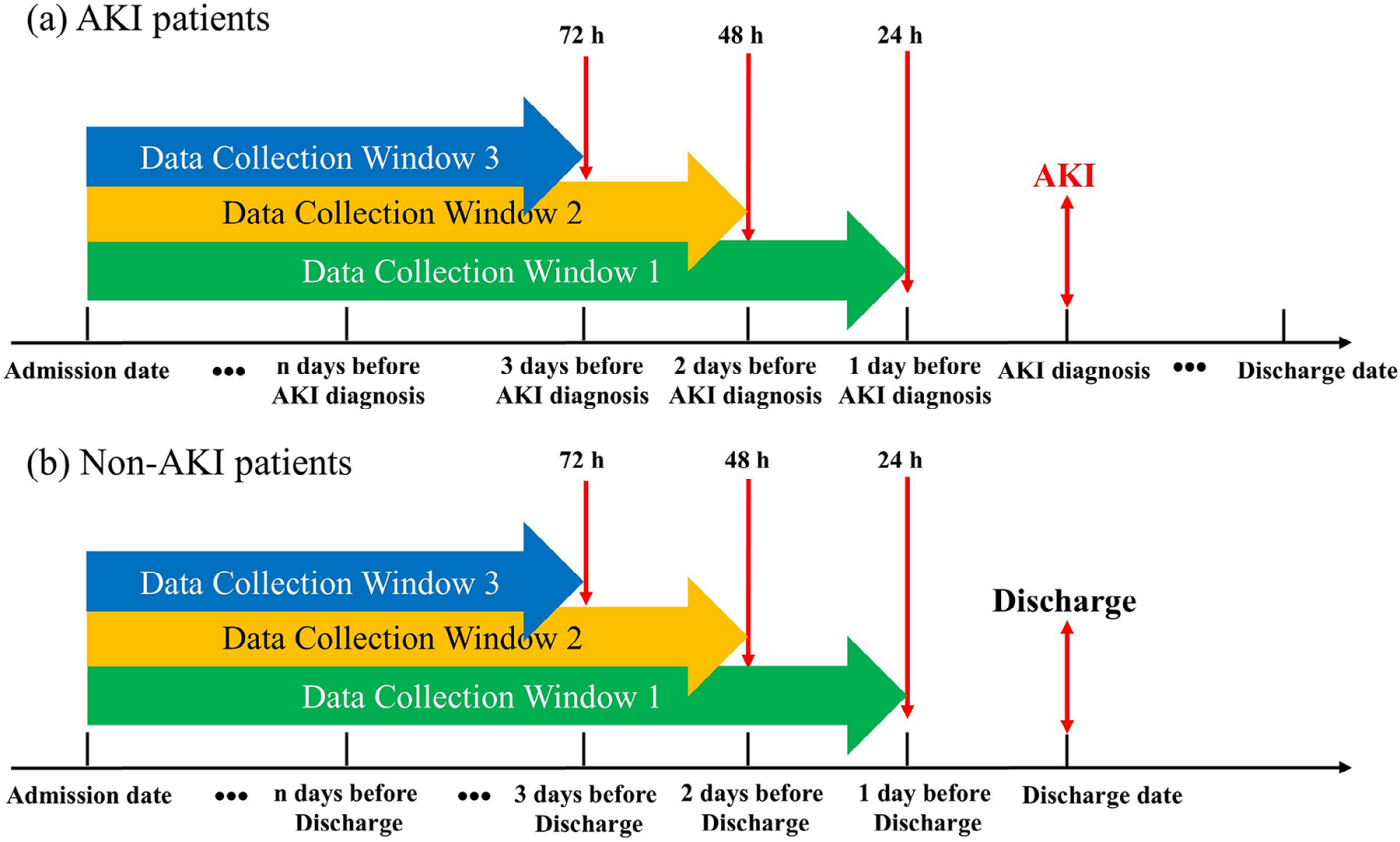
Date collection windows matching between the AKI patients and the non‐AKI patients. AKI, acute kidney injury.

The data collection windows for developing and validating AKI models were different between AKI patients and non‐AKI patients. For AKI patients, the data collection windows were between the admission date and 24, 48, or 72 h prior to the date of AKI identification using SCr levels. For non‐AKI patients, the data collection windows were between the admission date and 24, 48, or 72 h prior to the date of discharge (Figure 2).

### 2.4 Model construction workflow

**FIGURE 3:**
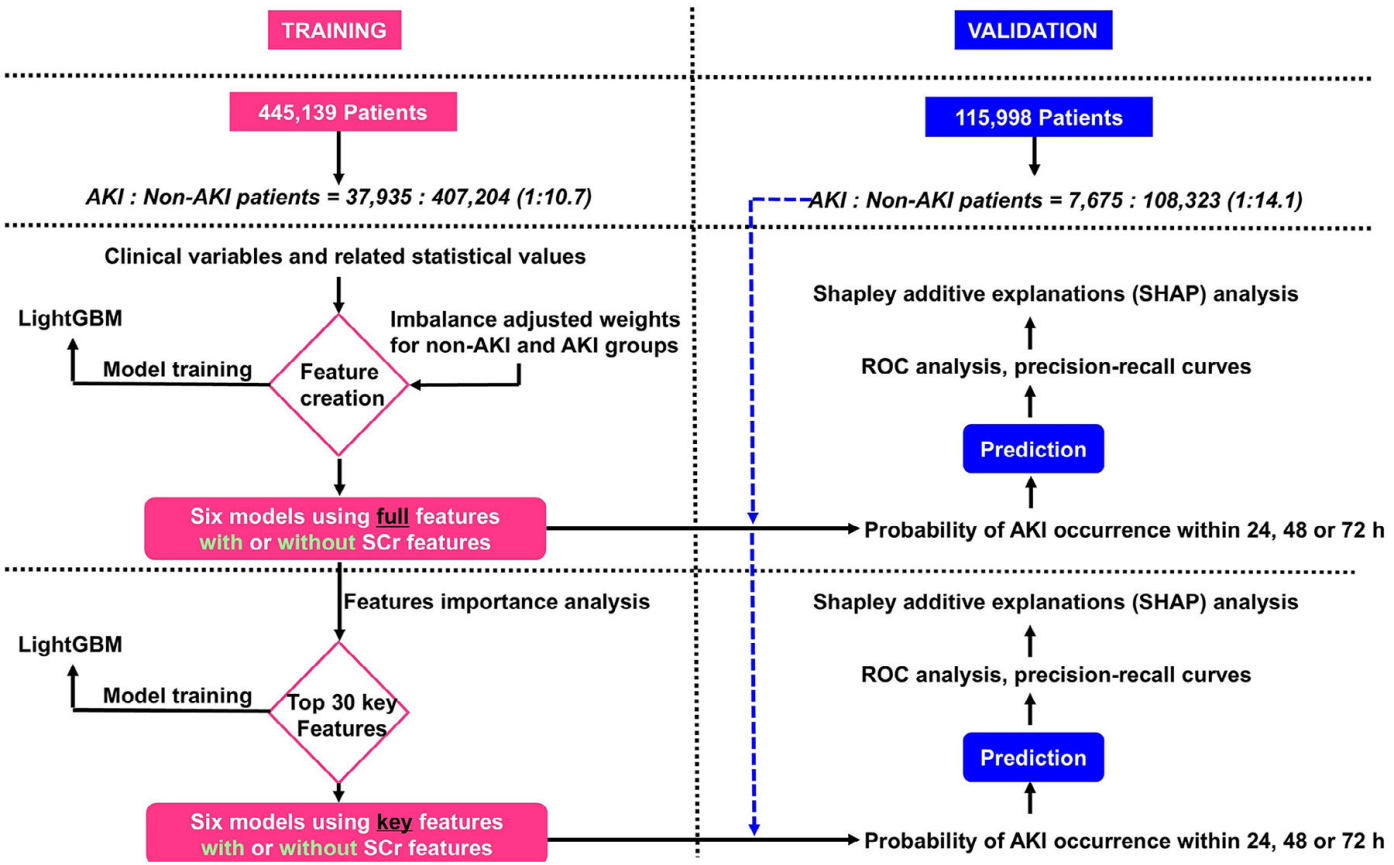
Model construction strategy and analysis workflow. AKI: acute kidney injury; LightGBM: light gradient boosting machine; ROC: receiver operating characteristic; SCr: serum creatinine.

Using data collected from the specified time windows, we aimed to predict whether a patient would develop AKI within the next 24, 48, or 72 h. For each prediction window, we utilized data from the corresponding collection window and developed models using the LightGBM classifier within the training cohort. As illustrated in Figure 3, a total of 12 models were constructed as follows:

1. Development of six models using full features: We began by training six initial models based on the full feature set. These included three models incorporating SCr features: one for each prediction window (24, 48, and 72 h), labeled as *24h_full_features_with_SCr*, *48h_full_features_with_SCr*, and *72h_full_features_with_SCr*. In parallel, we trained three corresponding models that excluded SCr features: *24h_full_features_without_SCr*, *48h_full_features_without_SCr*, and *72h_full_features_without_SCr*.
2. Feature ranking via feature importance analysis: For each of the six models, we conducted a feature importance analysis to assess the contribution of each feature to model performance. This was done using the LightGBM classifier's built‐in feature_importance function, which calculates importance based on the frequency with which a feature is used in split decisions across all trees. The top 30 features from each model were extracted to represent the most influential predictors.
3. Feature consolidation: We then consolidated the top features across models within each group. For the SCr‐inclusive models, we merged the three top‐30 feature sets (one from each time window) and removed duplicate entries, resulting in 55 unique features. A similar consolidation process was applied to the models without SCr, yielding 49 unique features after de‐duplication.
4. Development of six models using only key features: Using these refined feature sets, we retrained and evaluated a new set of six models—three with SCr and three without—across the 24, 48, and 72 h prediction windows. These final models, labeled *24h_key_features_with_SCr*, *48h_key_features_with_SCr*, *72h_key_features_with_SCr*, *24h_key_features_without_SCr*, *48h_key_features_without_SCr*, and *72h_key_features_without_SCr*, were developed using only the 55 or 49 selected features. In total, we developed and validated 12 predictive models.

### 2.5 Training Lightgbm classifier

The LightGBM classifier is a fast, distributed, and gradient‐boosting framework that uses tree‐based learning algorithms.^21^ It supports categorical features, handles missing data automatically, and adopts a leaf‐wise growth strategy with depth restriction within the decision tree. The LightGBM classifier generates a series of AKI decision trees, in which every tree optimizes the prediction results of previous trees.^22^, ^23^ Suppose Χ is the matrix of the patient's features inputted into the sub‐model $f_{i}\left(x\right)$ (i.e., a sub‐tree), then the composite model $F_{m}\left(X\right)$ is:

(1) $$F_{m}\left(X\right)=\partial_{0}\;f_{0}\left(X\right)+\partial_{1}f_{1}\left(X\right)+\cdots +\partial_{m}f_{m}\left(X\right)$$

where $\partial_{0}\cdots \partial_{m}$ are sub‐model coefficients, and *m* is the number of sub‐models. During the iterative process, the sub‐model was added one by one to the final model to ensure the loss function moving toward the gradient descent direction.

(2) $$L\left[F_{M}\left(X\right),Y\right]<L\left[F_{m-1}\left(X\right),Y\right]$$

where *L* is the loss function, *Y* is the output of AKI predictive model, that is, a probability of AKI occurrence in the next 24, 48, or 72 h. If the probability exceeded a chosen threshold (i.e., 0.5 in this study), a positive prediction would be made to trigger the AKI alert.

As there are only two classifications in this study (AKI vs. non‐AKI classes), the loss function is defined as

(3) $$\begin{array}{cc} binary\_logloss & =-\frac{1}{N}\sum_{i=1}^{N}(\mathrm{class}\_\mathrm{weight}\left[0\right]\times y_{i}\log p_{i}\\ & \;+\mathrm{class}\_\mathrm{weight}\left[1\right]\times \left(1-y_{i}\right)\log\left(1-p_{i}\right) \end{array}$$

where *N* is the total patient number in the training cohort used to train the LightGBM model, *y_i_* is the real classification of the input patient *i* having AKI, *1‐y_i_* is the real classification of the input patient *i* without AKI, *p_i_* is the output of the model to predict the probability of the input patient *i* having AKI, and *1‐p_i_* is the output of the model to predict the probability of the input patient *i* without AKI. And class_weight[0] and class_weight[1] are the weights for AKI patient and non‐AKI patient, respectively.

### 2.6 Tuning hyperparameters for optimizing LightGBM model performance

In our analysis using the LightGBM classifier to predict AKI, tuning hyperparameters was essential for optimizing model performance. We focused on three key hyperparameters: categorical features, class weight, and learning rate. The specific parameters governing the performance of the LightGBM classifier were optimized within the following predefined ranges: number of leaves (“num_leaves”, 31), maximum depth of binary tree (“max_depth”, ‐1) number of feature bins (“max_bin”, 255), number of iterations (“n_estimators”, 100), learning rate (“learning_rate”, 0.2), device type (“device type”, CPU), categorical feature (“gender”, “hospital department”, “hospital grade”), and class weight.

Categorical feature optimization: We harnessed LightGBM's histogram‐based optimization for categorical features. Variables such as ‘gender’, ‘hospital department’, and ‘hospital grade’ were designated as categorical, enhancing the model's ability to effectively leverage these features for improved prediction accuracy.

Class weight adjustment: In machine learning, training on an imbalanced dataset can cause the classifier to favor the majority class, resulting in biased predictions for the minority class—AKI patients in this case. To combat the data imbalance between AKI and non‐AKI groups, we adjusted the ‘class weight’ hyperparameter.

We assigned larger weights to AKI patients' data compared to non‐AKI patients' data, applying iterative calibration to improve prediction accuracy. Specifically, the weight for the non‐AKI class was fixed at 1, while the weight of the AKI class was adjusted from 1 to 12. Various weight combinations (e.g., 1:1, 1:2, 1:3, …, 1:12) were individually inputted into the LightGBM classifier along with the constructed features. Based on the performance of the AKI predictive models, the optimal class weights for AKI and non‐AKI cases were determined.

Learning rate tuning: The learning rate is crucial in LightGBM, affecting the model's update size during learning. We applied a methodical optimization strategy, adjusting the learning rate in 0.05 increments from 0 to 0.5. The selected rate was the one that achieved the highest F1 score on the validation set, which led to an optimal learning rate between 0.1 and 0.25 for different models.

The threshold of LightGBM classifier was set at 0.5. When the output of LightGBM classifier was equal to or larger than 0.5, the AKI model predicted that the patient will be developing AKI in the next 24, 48, or 72 h; and when the output was smaller than 0.5, the AKI model predicted that the patient will not have AKI in next 24, 48, or 72 h. This rigorous approach to hyperparameter tuning significantly enhanced the robustness and generalizability of our AKI predictive models. By fine‐tuning the handling of categorical features, class weights, and learning rates, we ensured our models' optimal performance across a variety of scenarios and data distributions.

### 2.7 Shapley additive explanations (SHAP) analysis

To further analyze the positive and negative influences of features, which were used to build the model predictions, Shapley additive explanations (SHAP) analysis was conducted in the independent test cohort. The SHAP value quantifies each feature's contribution to an individual prediction, indicating both the magnitude and direction of its impact. Thus, the SHAP value definition is as follows:

(4) $$y_{i}=y_{base}+f\left(x_{i,1}\right)+f\left(x_{i,2}\right)\cdots +f\left(x_{i,k}\right)$$

where $y_{i}$ is the predicted value outputted from LightGBM classifier for the single data *i* in the test cohort; $y_{base}$ as the base value is the mean of the model output over the training cohort; $x_{i,j}$ is the feature *j* of the single data *i*; $f(x_{i,j})$ is the SHAP value for feature $x_{i,j}$. The SHAP feature value $f(x_{i,j})$ represents the feature $x_{i,j}$ contributed toward the prediction value $y_{i}$. If $f(x_{i,j})$ > 0, this feature $x_{i,j}$ has a positive contribution to the prediction value $y_{i}$; while $f(x_{i,j})$ < 0, this feature $x_{i,j}$ has a negative contribution to the prediction value $y_{i}$.

### 2.8 Model performance evaluation and statistical analysis

To evaluate the prediction performance of the developed AKI models, five metrics, including precision, recall (sensitivity), specificity, accuracy, and area under the curve (AUC) were calculated from the receiver operating characteristic curve (ROC) using the model outputs from the independent test cohort:

$$\mathrm{precision}=\frac{\mathrm{TP}}{\mathrm{TP}+\mathrm{FP}}$$

$$\mathrm{recall}=\frac{\mathrm{TP}}{\mathrm{TP}+\mathrm{FN}}$$

$$\mathrm{specificity}=\frac{\mathrm{TN}}{\mathrm{TN}+\mathrm{FP}}$$

$$\mathrm{accuracy}=\frac{\mathrm{TP}+\mathrm{TN}}{\mathrm{TP}+\mathrm{TN}+\mathrm{FP}+\mathrm{FN}}$$

where TP, TN, FP, and FN represent true positive, true negative, false positive, and false negative, respectively. AUC represents the classifier's ability to distinguish between positive and negative classes.

The 10‐fold cross‐validation was applied to internally validate the overall performance and acquired optimal parameters of all prediction models using the training set. The performance of the developed models was also validated using an independent test cohort.

For numeric variables, mean and standard deviation were calculated, and the differences between AKI and non‐AKI patient groups were compared using rank‐sum tests. For categorical variables, occurrences and percentages were shown, and the difference between the training cohort and independent test cohort was compared using chi‐square tests. Differences between groups were considered statistically significant if *p* < 0.05.

This study was registered at ClinicalTrials.gov (Registration Number: NCT03061786). The study protocol complied with the Declaration of Helsinki and was approved by the Guangdong Provincial People's Hospital Institutional Review Board (IRB# GDREC.2016327H). The informed consent was waived given the retrospective nature of the study.

## 3 RESULTS

### 3.1 Clinical feature characteristics of patient data

Baseline characteristics are provided in Table S5, which details the demographic and clinical variable distributions for both AKI and non‐AKI patients. Table S6 summarizes the clinical features of patient data used in the training and independent test cohorts. Differences in variable distributions were observed between the independent test cohort and the training cohort, likely due to the test cohort being sourced from different hospitals. These differences offer a meaningful basis for assessing model robustness and generalizability (Table S6).

**FIGURE 4:**
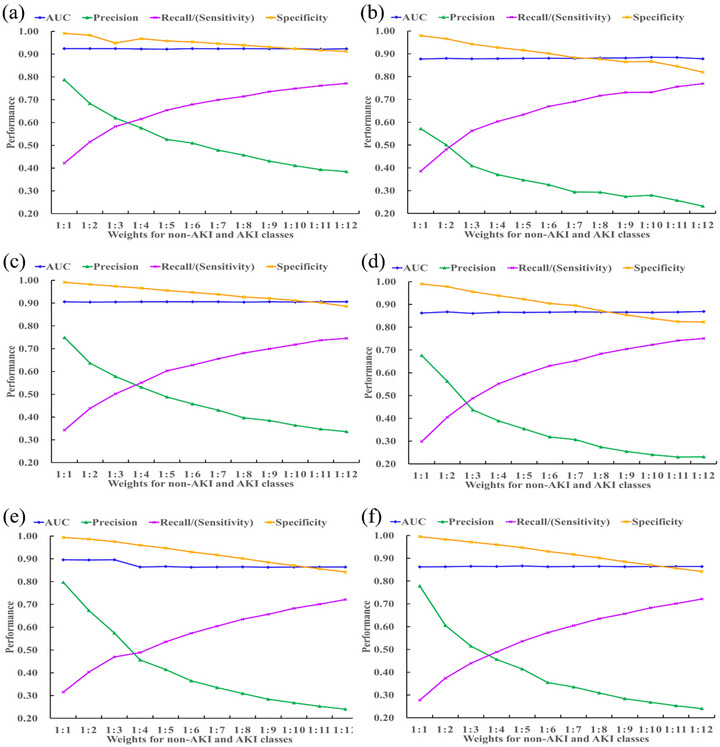
Prediction performance comparison of LightGBM classifiers using different class weights for non‐AKI and AKI classes. The prediction performance of AKI model with SCr features using different class weights for non‐AKI and AKI classes to predict the occurrence of AKI in next 24 (a), 48 (c) and 72 h (e). The prediction performance of AKI model without SCr features using different class weights for non‐AKI and AKI classes to predict the occurrence of AKI in next 24 (b), 48 (d), and 72 h (f). AKI, acute kidney injury.

With imbalance adjustment of class weights between AKI and non‐AKI patients performed, Figure 4 illustrates that the AKI predictive models, with or without using SCr features, achieved optimal performances when the class weights for non‐AKI and AKI classes were set to 1:3.

### 3.2 Performance of AKI predictive models using full features

Table 1 presents the prediction performance results of models using full features to predict the AKI occurrence in the test cohort within the next 24, 48, and 72 h. The AUC, specificity, and accuracy of the AKI prediction for the next 24–72 h reached high values ranging from 0.861 to 0.986. Moreover, the AKI model without using SCr features still achieved strong prediction performance.

**TABLE 1:** Prediction performances of AKI models using full features.

AKI Models with or without SCr features included	Features number included	AUC	Precision	Sensitivity (Recall)	Specificity	Accuracy
24h_full_features_with_SCr	107	0.925 (0.921,0.923)	0.684 (0.681,0.687)	0.515 (0.504,0.526)	0.983 (0.982,0.984)	0.952 (0.951,0.953)
24h_full_features_without_SCr	100	0.878 (0.873, 0.883)	0.409 (0.406,0.412)	0.563 (0.552,0.574)	0.942 (0.982,0.984)	0.917 (0.915,0.919)
48h_full_features_with_SCr	107	0.905 (0.901, 0.910)	0.637 (0.634,0.640)	0.438 (0.427,0.449)	0.982 (0.981,0.983)	0.946 (0.945,0.947)
48h_full_features_without_SCr	100	0.861 (0.856, 0.866)	0.437 (0.434,0.440)	0.488 (0.477,0.499)	0.955 (0.954,0.956)	0.925 (0.924,0.926)
72h_full_features_with_SCr	107	0.895 (0.890, 0.900)	0.673 (0.670,0.676)	0.403 (0.392,0.414)	0.986 (0.985,0.987)	0.949 (0.948,0.950)
72h_full_features_without_SCr	100	0.865 (0.860, 0.870)	0.515 (0.512,0.518)	0.440 (0.429,0.451)	0.971 (0.970,0.972)	0.937 (0.936,0.938)

The range of values in parentheses indicates the 95% confidence interval.

Abbreviations: AKI, acute kidney injury; AUC: Area under the curve; SCr: serum creatinine.

**FIGURE 5:**
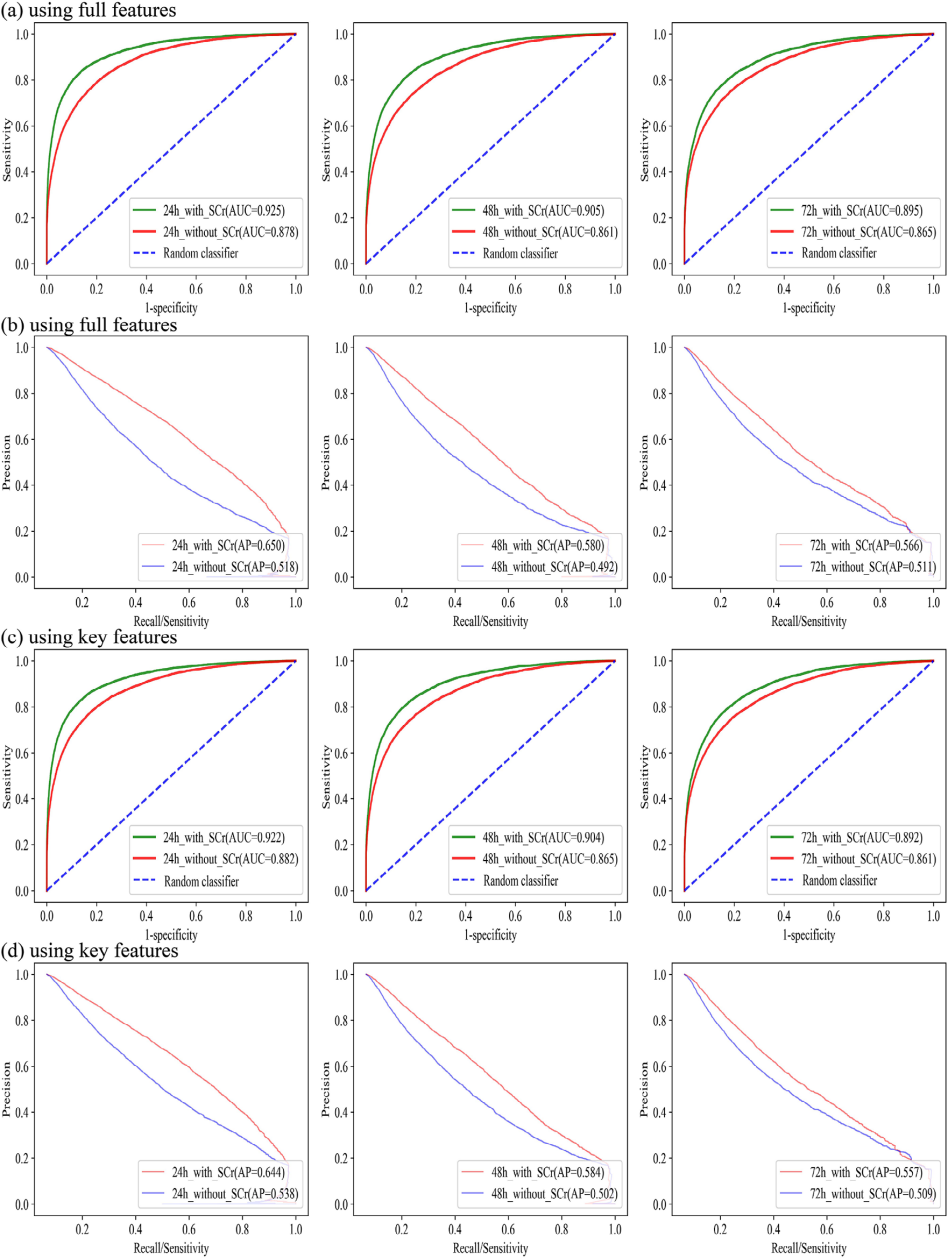
Performance of AKI Models using full features and key features presented by receiver operating curve (a,c) and precision‐recall curves (b, d) within next 24, 48, and 72 h. AP, average precision; AUC, area under the curve; SCr, serum creatinine.

Figure 5a,b displays the corresponding ROC and precision‐recall curves for the AKI models using full features, with or without SCr features included, to predict the occurrence of AKI in the next 24, 48, and 72 h. The ROC corresponds to the AUC value, and the precision‐recall curve corresponds to the value of average precision (AP). The models showed robust predictive performance for AKI regardless of whether SCr features were included.

### 3.3 An example of AKI risk prediction using developed models

**FIGURE 6:**
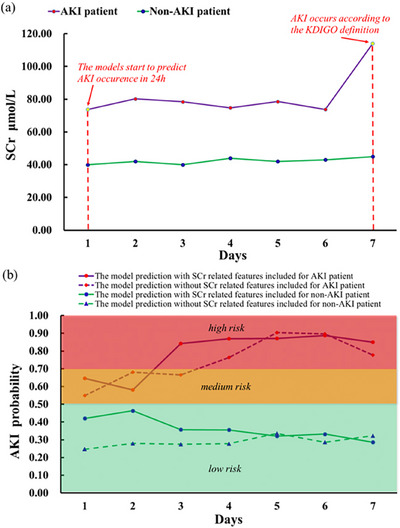
An example of AKI risk prediction. Every 24 h and corresponding changes of SCr levels in two patients during a 7‐day prediction window, one female patient aged 80 with peripheral vascular disease (AKI patient) and cerebrovascular disease; and the other male patient aged 46 admitted to intensive care unit (non‐AKI patient). (a) The longitudinal changes of SCr levels. (b) AKI risk predictions every 24 h during the 7‐day prediction window. AKI, acute kidney injury; SCr, serum creatinine.

To demonstrate the clinical utility of the developed AKI models, Figure 6 serves as an example by illustrating the models' application to two inpatients. Figure 6a displays the longitudinal changes in SCr levels and the continuous predictions of the AKI models (with or without SCr features) concerning the probability of AKI occurrence within the next 24 h (Figure 6b) for each day during a 7‐day period window for two patients.

Figure 6a reveals that one patient's SCr levels remained stable, ranging from 40 to 45 µmol/L, while the other patient's SCr levels fluctuated between 73.8 and 80.2 µmol/L during the first 6 days before rapidly increasing to 113.9 µmol/L on day 7, when this patient was diagnosed with AKI according to KDIGO criteria.

For the AKI patient, the proposed AKI models could continuously predict AKI occurrence within 24 h with a probability of 0.55 (medium risk) without SCr features and 0.65 (medium risk) with SCr features on the first 24 h after hospital admission. Although the model without SCr features predicted moderate risk on day 2, it rose to high risk on day 3 and continued this increasing trend until day 7 (Figure 6b). The model with SCr features consistently predicted AKI occurrence with high risk during the 7‐day period window. On day 7, AKI was diagnosed via SCr measurement for this patient. The developed models, both using and not using SCr features, could predict the risk as early as 6 days prior to the AKI occurrence.

In contrast, the AKI models predicted that the patient with stable SCr levels had low AKI risk, with a probability of less than 0.42 during the 7‐day period window. Furthermore, the AKI model without SCr‐related features included could still achieve comparable performance in risk estimation for these two patients.

In the prediction results, models with and without SCr features showed differences, with the model excluding SCr generally predicting lower probabilities. Despite this difference in prediction probabilities, their ability to assess risk is very close. Particularly for the patient clinically confirmed with AKI on the seventh day, the model was able to predict a medium risk of AKI on the day of admission, regardless of whether SCr‐related features were used. This indicates that the model remains reliable even without SCr data.

**FIGURE 7:**
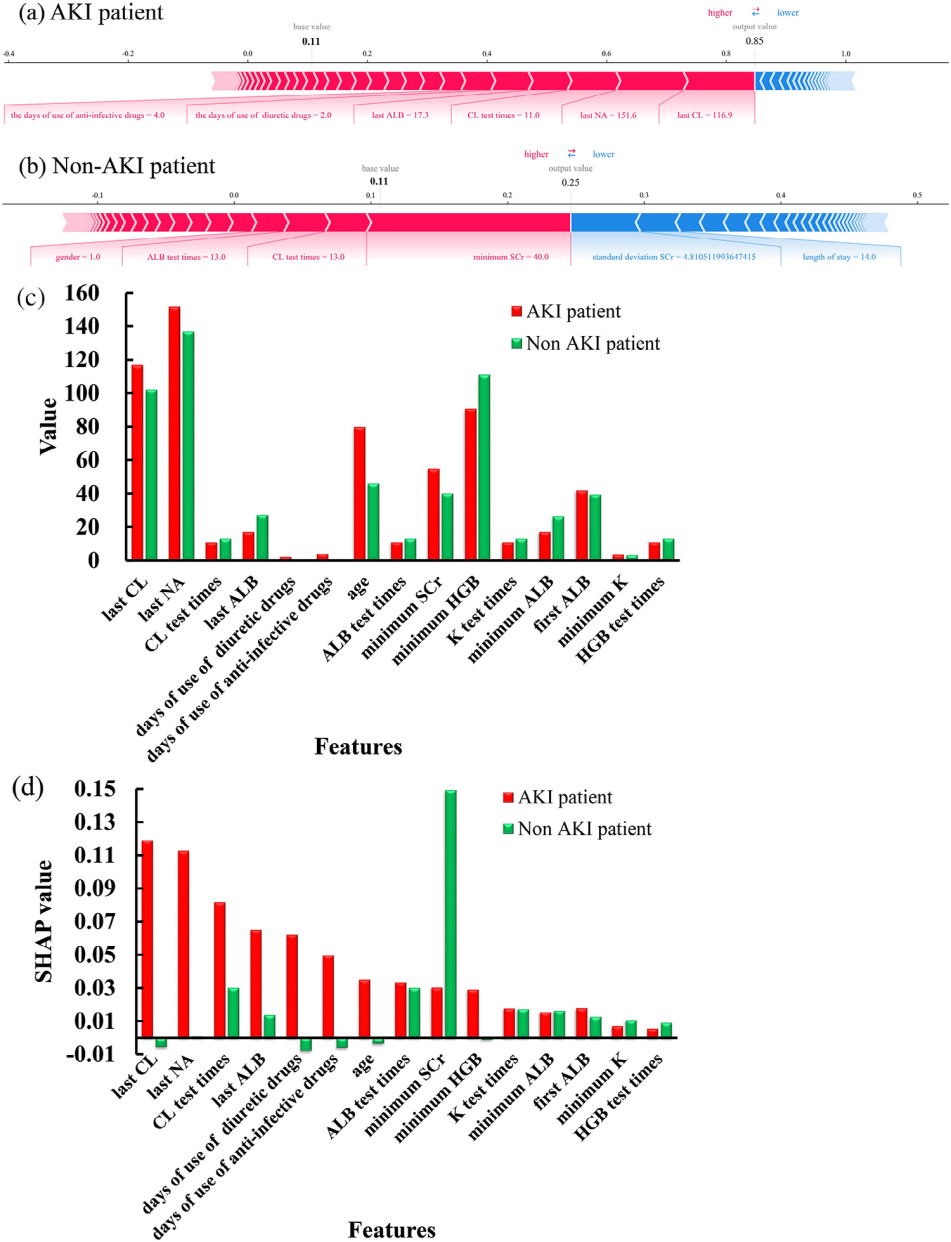
The SHAP values of features from two example patients (same patients in Figure 6) using AKI model with full features including SCr features to predict the occurrence of AKI in next 24 h. The SHAP analysis of the AKI patient (a) and the non‐AKI patient (b), the first 15 SHAP values of the AKI patient (c) and the non‐AKI patient (d). AKI, acute kidney injury; ALB, albumin; Cl, chloride; Na, sodium; SCr, serum creatinine; HGB, hemoglobin; K, potassium.

Figure 7 presents an example of the SHAP values of features from the same two patients depicted in Figure 6. In Figure 7, the base value of 0.11 is the mean of the fitting values of targets in the training cohort. The red bar indicates a positive contribution, while the blue bar indicates a negative contribution. Each segment of the blue or red arrow bar represents a single feature with its individual SHAP feature value contributing to the final prediction value (e.g., for the AKI patient, the feature value of the last chloride (CL) value = 116.9 mmol/L, the SHAP value of the last CL value = 0.12; The feature value of the last sodium (Na) value = 151.6 mmol/L, the SHAP value of the last Na value = 0.11, etc.). Longer red or blue bars have a larger positive or negative impact on the final prediction value.

Figure 7c,d displays the first 15 SHAP values features, including the top 10 SHAP values of the AKI patient in Figure 7a and the top 10 SHAP values of the non‐AKI patient in Figure 7b, along with five overlapping features between AKI and non‐AKI patients. For the AKI patient, the top 10 features include the last chloride (CL) value, last sodium (Na) value, CL test times, last albumin (ALB) value, days of diuretic drug use, days of anti‐infective drug use, age, ALB test times, minimum SCr value, and minimum hemoglobin (HGB) value.

CL is an electrolyte that helps maintain the balance of body fluids and the body's acid–base balance. The AKI patient's last CL value (116.9 mmol/L) exceeded the normal range of 98–106 mmol/L. Similarly, the last Na value (151.6 mmol/L) was beyond the normal range of 135–145 mmol/L. Moreover, the last ALB value of 17.3 g/L, as the minimum value, is far below the normal range of 35–50 g/L, and the minimum HGB value of 91 g/L is also below the normal range (110–150 g/L). Additionally, the CL test times (11), minimum SCr value (55 µmol/L), days of diuretic drug use (2), days of anti‐infective drug use (4), and age (80 years) also had significant positive effects on the prediction performance of the developed AKI model using full features with SCr features included. These top features play a crucial role in predicting AKI occurrence, especially abnormal laboratory measurements related to metabolism and renal dysfunction, which are key factors in determining the risk of AKI onset.

For the non‐AKI patient, the top 10 features that contributed to the prediction included the minimum SCr value, CL test times, ALB test times, gender, *K* test times, the minimum ALB value, the last ALB value, the first ALB value, the minimum *K* value, and HGB test times. During the 7‐day period, this patient maintained low and stable SCr levels and had a lower minimum SCr level (40 µmol/L) than the AKI patient (55 µmol/L). The patient also underwent 13 CL, ALB, *K*, and HGB laboratory tests. The minimum ALB value of 26.2 g/L and the last ALB value of 27.2 g/L were slightly below the normal range. However, the first ALB value of 39.4 g/L, the minimum K value of 3.03 mmol/L, and the minimum HGB value of 111 g/L were within the normal ranges. This patient was in a stable condition without AKI risk, which could be attributed to the normal and stable levels of most clinical measurements.

The non‐AKI patient's stable condition and low risk of AKI could be linked to the normal and stable levels of most clinical measurements. The AKI prediction model, which took into account the top features for each patient, was able to distinguish between the AKI and non‐AKI patients and provide a reliable risk assessment for both patients. This predictive capacity of our models, offering advanced warning days before clinical diagnosis, underscores their potential value in alerting healthcare providers early. Such early detection could enable timely interventions, significantly enhancing patient outcomes.

### 3.4 Feature importance analysis

**FIGURE 8:**
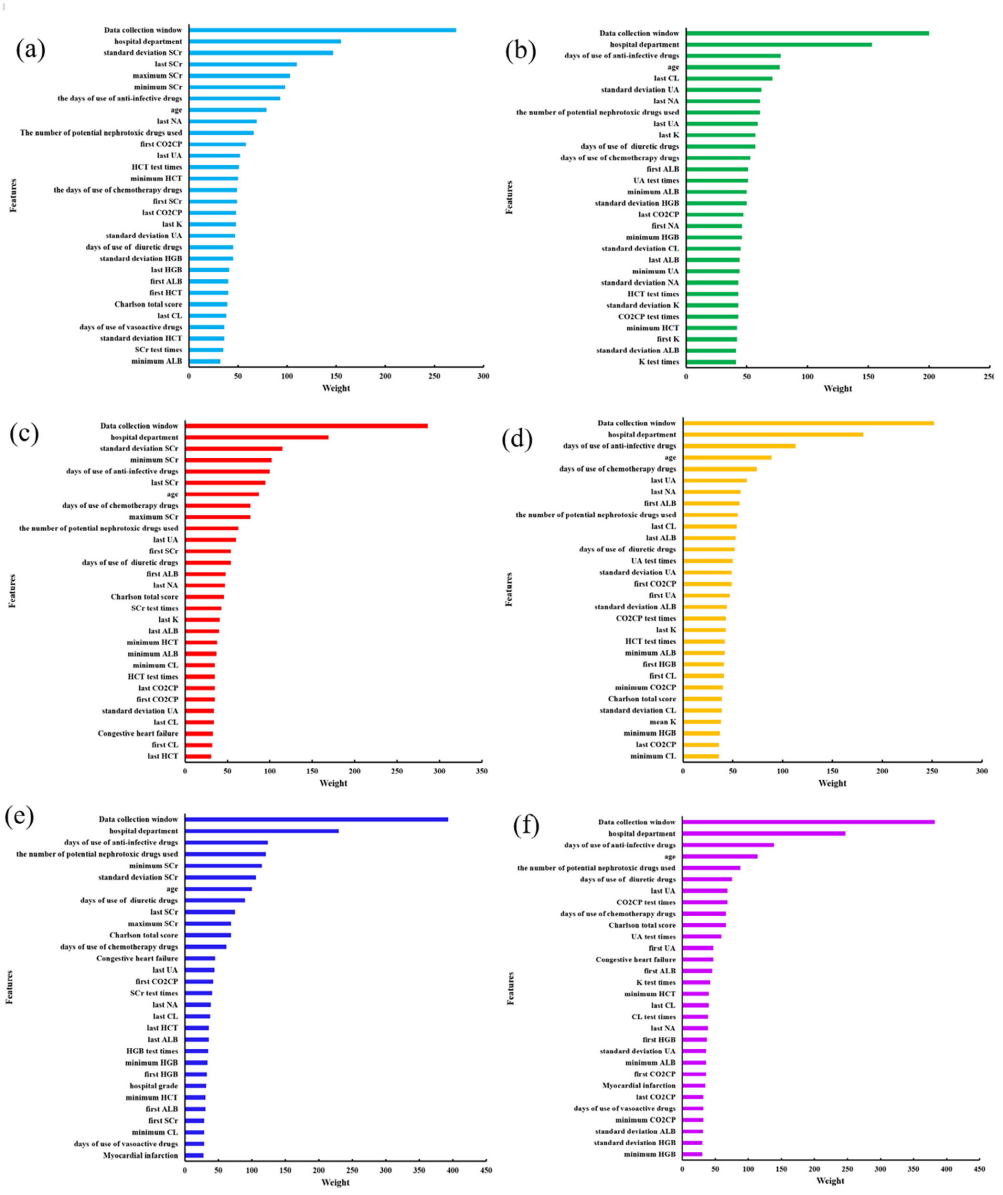
Weights for top 30 dominant features in different AKI models with different features included. (a) 24 h with SCr features; (b) 24 h without SCr features; (c) 48 h with SCr features; (d) 48 h without SCr features; (e) 72 h with SCr features; (f) 72 h without SCr features. AKI, acute kidney injury; SCr, serum creatinine.

Figure 8 and Table S7 present the feature importance analysis, ranking the top 30 features according to their contribution to the predictive performance of the AKI models developed using full features.

### 3.5 Performance of AKI predictive models using only key features

Table 2 presents the performance of models using only key features across different prediction windows, with or without the inclusion of SCr features. Figure 5c–d illustrates the corresponding ROC and precision‐recall curves. When SCr features were included, the models showed strong predictive performance for AKI, with most accuracy, specificity, and AUC values exceeding 0.9. Even without SCr‐related features, the models still performed well. Sensitivity values were comparatively lower, reflecting potential limitations in identifying true AKI cases—primarily due to the small number of AKI events and the model's bias toward non‐AKI patients.

**TABLE 2:** Prediction performances of AKI models using key features.

AKI Models with or without SCr features included	Features number included	AUC	Precision	Sensitivity (Recall)	Specificity	Accuracy
24h_key_features_with_SCr	55	0.922 (0.918, 0.926)	0.609 (0.606,0.612)	0.584 (0.573, 0.595)	0.973 (0.972, 0.974)	0.948 (0.951, 0.953)
24h_key_features_without_SCr	49	0.882 (0.877, 0.887)	0.452 (0.449,0.455)	0.550 (0.539, 0.561)	0.953 (0.952, 0.954)	0.926 (0.915, 0.919)
48h_key_features_with_SCr	55	0.904 (0.899, 0.909)	0.578 (0.575,0.581)	0.510 (0.499, 0.521)	0.974 (0.973, 0.975)	0.943 (0.945, 0.947)
48h_key_features_without_SCr	49	0.865 (0.860, 0.870)	0.457 (0.454,0.460)	0.486 (0.475, 0.497)	0.959 (0.958, 0.960)	0.928 (0.924, 0.927)
72h_key_features_with_SCr	55	0.892 (0.887, 0.897)	0.554 (0.551,0.557)	0.485 (0.474, 0.496)	0.973 (0.972, 0.974)	0.941 (0.948, 0.950)
72h_key_features_without_SCr	49	0.861 (0.856, 0.866)	0.504 (0.501,0.507)	0.450 (0.439, 0.461)	0.969 (0.968, 0.970)	0.936 (0.936, 0.938)

The range of values in parentheses indicates the 95% confidence interval.

Abbreviations: AKI, acute kidney injury; AUC, area under the curve; SCr, serum creatinine.

### 3.6 Relationship between admission departments and AKI incidence rate

**FIGURE 9:**
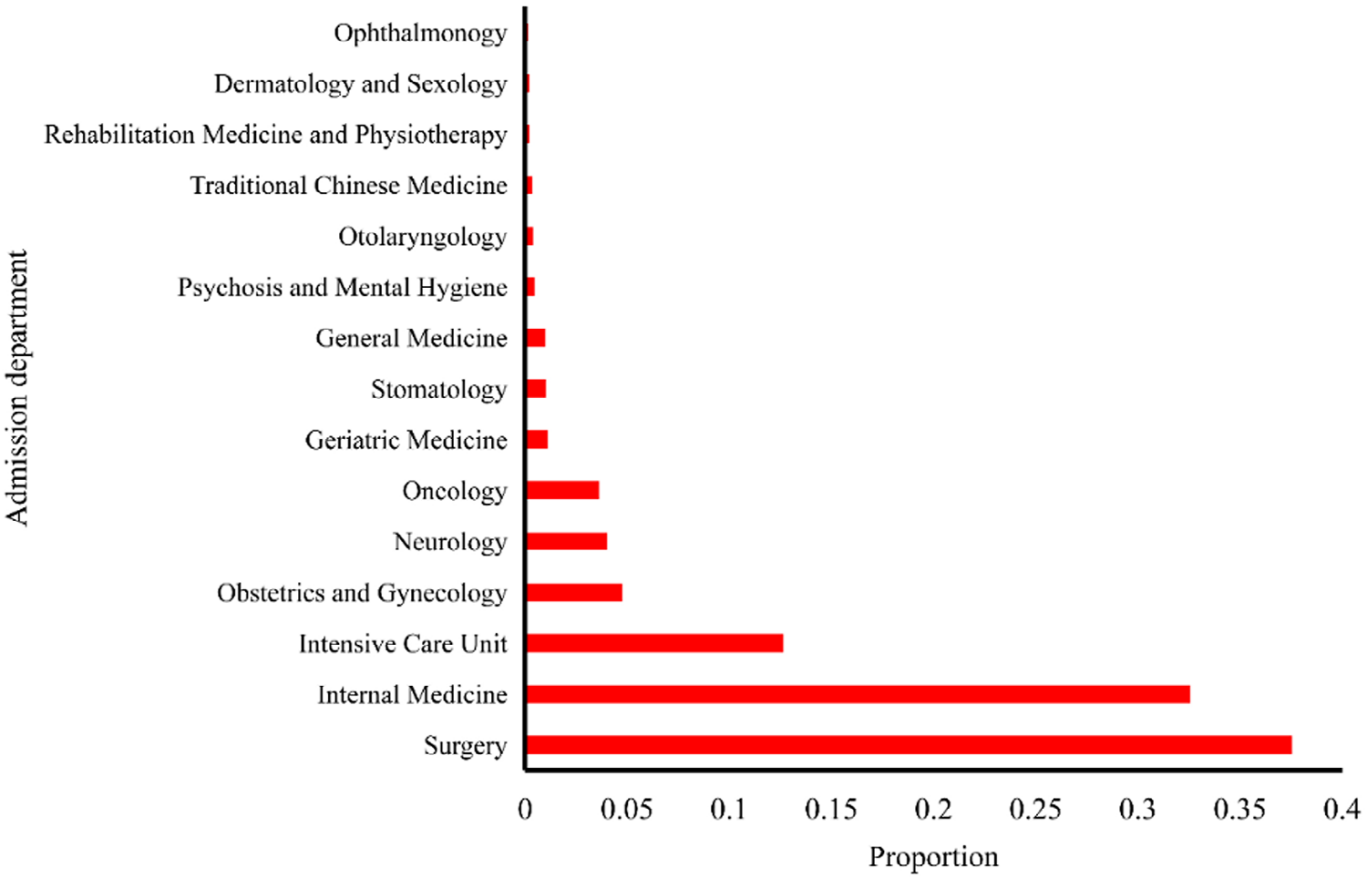
Proportion of admission departments for acute kidney injury inpatients.

**FIGURE 10:**
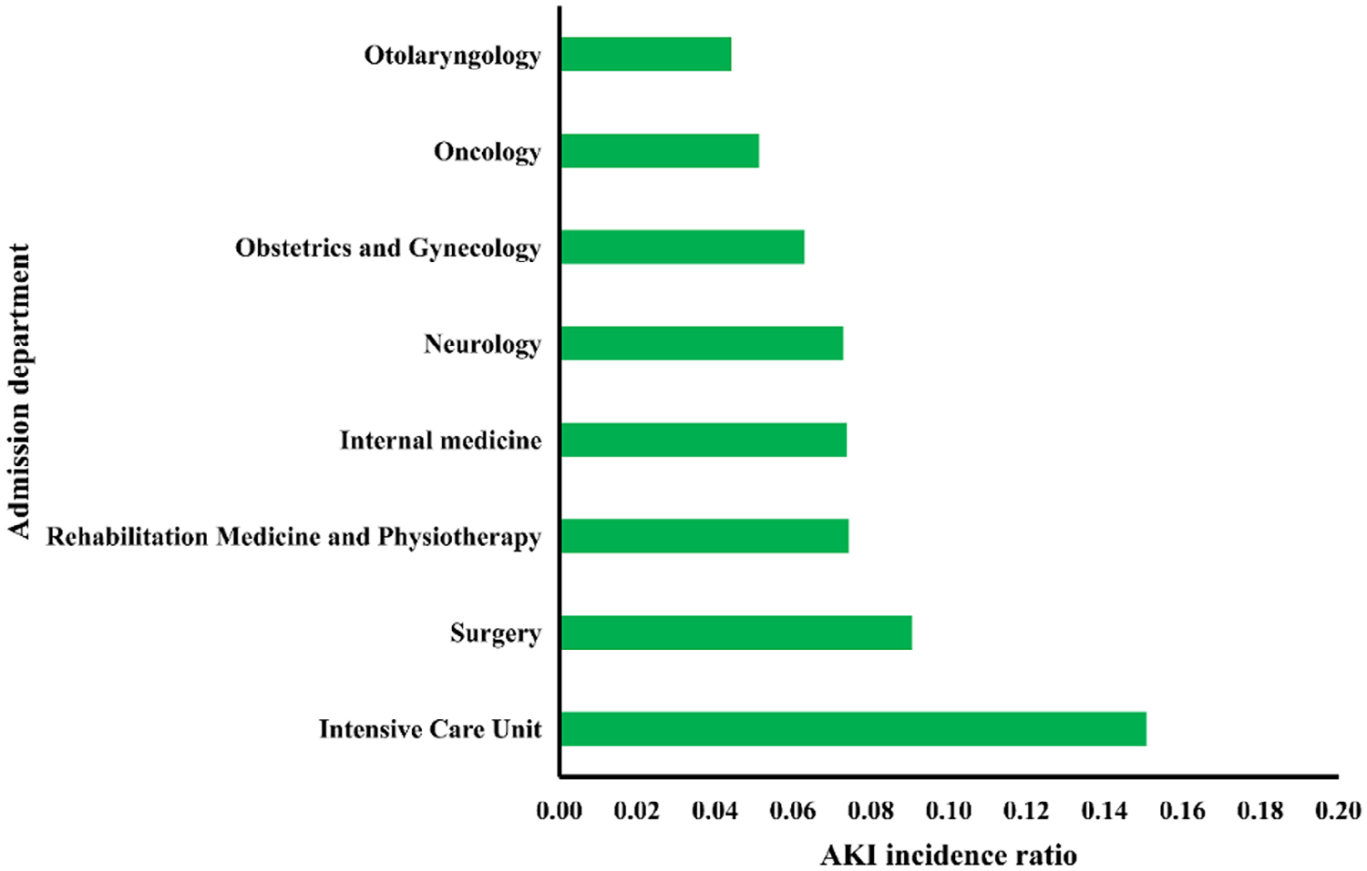
Acute kidney injury incidence ratio in admission departments. AKI: acute kidney injury.

Regarding admission departments, inpatients in different departments often undergo various examinations and treatments. Their proportions and AKI incidence rate in each department are shown in Figures 9 and 10. According to patient enrollment, most of the hospitalized patients who developed AKI were admitted into six departments: intensive care unit, surgery, rehabilitation medicine and physiotherapy, internal medicine, neurology, oncology, and otolaryngology department (Figure 10). The inpatients in these departments had higher AKI incidence risks, and the eight departments possess the top eight AKI incidence rates.

## 4 DISCUSSION

This study addresses several significant challenges in the development of machine learning‐based AKI models for LMICs, including inconsistent SCr measurements, non‐standard EMRs, validation of developed models using real‐world datasets from resource‐limited settings, and identifying the most critical features from non‐standard EMRs that are indicative of AKI risk. The study successfully leverages a large‐scale dataset from 15 hospitals with varying resources, representative of the current healthcare landscape in China, to develop prediction models. These models demonstrate promising performance, serving as a paradigm for creating disease models in LMICs by employing non‐standardized EMRs combined with data preprocessing techniques.

In many LMICs settings, EMR systems lack uniform data structures, standardized field names, and consistent recording practices (i.e., non‐standard EMRs). Our non‐standard EMR data were derived from the most developed to poorly developed regions in China. As a result of the essential data preprocessing, we used the LightGBM classifier to construct models, which can achieve promising overall performance comparable to the studies reported previously that required thousands of feature dimensions based on standardized EMR.^11^, ^12^, ^13^ The comparable prediction performances of our model indicated that essential information can be reserved through non‐standard data preconditioning, which can provide a clinically translatable solution of using the LightGBM‐enabled AKI models in LMICs.

The outstanding performance of the developed algorithms can be primarily attributed to the unique advantages of LightGBM. In contrast to traditional level‐wise generation methods, LightGBM employs a leaf‐wise generation strategy, which facilitates integration into parallel learning, consequently reducing training errors and enhancing the accuracy of the surrogate model. To prevent overfitting during AKI model development, LightGBM offers a parameter to limit the decision tree's depth. The default value for the maximum depth of the binary tree (i.e., the max_depth parameter) is set to ‐1, signifying no limit. Simultaneously, the maximum number of leaves in a single tree is determined by the num_leaves parameter, which should be set to less than two times the max_depth to circumvent overfitting. Given the 107‐feature dimension in this study, the number of leaves and maximum depth of the binary tree were assigned their default values of 31 and −1, respectively.

Additionally, LightGBM employs a histogram‐based algorithm to accelerate training and decrease memory usage. This method replaces continuous values with discrete bins and searches for the optimal segmentation point, eliminating the need to store extra information for pre‐sorting feature values. The max_bin parameter defines the maximum number of bins into which feature values will be grouped. A smaller number of bins may decrease training accuracy but potentially increase the ability to mitigate overfitting. In this study, the default max_bin value was set to 255, and LightGBM utilized compressed 8‐bit unsigned integers for feature values.

We also tested models built using only the top 10 features from each full‐feature model. Although these “top‐10” models maintained reasonable discriminatory ability, they showed a noticeable drop in performance—reflected by an average AUC reduction of approximately 0.02 and consistently lower net benefit across most decision thresholds. This finding underscores the value of features ranked 11 to 30, which still contribute meaningful predictive information. Therefore, our selection of 49 and 55 features represents a deliberate balance between model parsimony and predictive performance.

In this investigation, we confronted the predictive challenges of AKI due to EMR inconsistencies. Our CCS‐AKI database, reflective of these irregularities, often lacks comprehensive medical data and essential vital signs.^5^, ^7^, ^24^ This is in stark contrast to wealthier regions where EMRs are replete with data, including SCr levels—a cornerstone for AI‐predictive models of AKI, as demonstrated by DeepMind and similar initiatives.^13^, ^25^

For AKI diagnosis, clinical guidelines mandate a minimum of two SCr tests within a week. However, the primary goal of our model is to anticipate AKI risk before its onset, thus facilitating timely interventions that may reduce its severity. Although SCr analysis is cost‐effective and fundamental for diagnosis, it is not an early AKI indicator, often lagging in detection.^26^

Regarding the importance of feature design in AKI prediction, the most critical factor was the data collection window. In this study, the data collection window spanned from admission until AKI identification (for AKI patients) or discharge (for non‐AKI patients). We found that the model using a 24‐h window prior to AKI onset achieved superior predictive performance compared to the 72‐h window. This likely reflects the tendency for AKI to occur early during hospitalization. The improved performance of the 24‐h model can be attributed to its temporal proximity to the AKI event, which enables the capture of acute physiological changes critical for accurate prediction.

The second most important feature was the admission department, representing demographic characteristics, pre‐existing diseases, and medications used. Additionally, renal function declines with age, so age was also included in the key features ranking list. Common drugs associated with kidney injury, such as antibiotics, diuretics, and chemotherapy, played significant roles in AKI prediction.

Our models consider other biochemical markers, such as electrolyte balances, renal function‐related biochemical and hematological indicators, commonly included in routine blood tests and available within EMRs. Fluctuations in sodium, potassium, chloride, and uric acid levels in the blood reflect renal conditions, as AKI can lead to fluid imbalance and electrolyte disorders,^27^ corresponding to hyponatremia and hyperkalemia. Incorporating these parameters, even in models excluding SCr features, harnesses the information‐rich routine blood work, enabling effective prediction in settings where SCr is less frequently tested. By capitalizing on routine clinical data, our approach adheres to a “zero‐cost” paradigm for predicting AKI risk, especially beneficial in resource‐scarce settings.

This study acknowledges multiple limitations. First, the lack of repeated SCr tests led to the exclusion of a significant portion of the patient cohort, raising the possibility of selection bias. The SCr‐based KDIGO criteria were considered the “gold standard for AKI.” Due to the retrospective nature of the CCS‐AKI data and the limited frequency of SCr tests, undetected AKI cases were inevitable in the current situation in China. Consequently, a future prospective clinical trial is necessary to validate the utility of the developed AKI models.

Second, the study presents a unified model that amalgamates data from all clinical settings, integrating data from various clinical contexts into a single model could sometimes diminish its efficacy. Nonetheless, this strategy broadens the applicability of our AKI prediction models. A model that achieves a balance between generalizability and predictive accuracy is more clinically valuable. Our research prioritizes the models' transferability and adaptability across diverse clinical landscapes.

Our model employs data from non‐standardized EMRs, utilizing a set of widely available indicators, which serves as a foundation for creating a universally applicable AKI prediction model. The model's consistent predictive value during training and testing suggests the viability of using non‐standardized EMR data to create an effective prediction tool.

Although specialized models may offer enhanced precision within their respective clinical domains, they often demand more complex data, which may not be readily available in non‐standardized EMRs, limiting their practical application. Our focus on model generalizability and adaptability does not overlook the impact of clinical context on performance. Future work will assess the model's versatility in different settings, factoring in demographics, comorbidities, and treatment protocols. We may explore refining the model for specific environments, potentially through parameter adjustments, inclusion of context‐specific variables, or the development of tailored models for unique clinical situations. Such targeted improvements are expected to improve the model's accuracy and utility across various clinical contexts.

Third, our study's cohorts were exclusively from China, which may raise concerns of limiting the generalizability of our findings due to the variation in EMRs across different healthcare systems and practices globally. Nonetheless, the hospitals in our Chinese CCS‐AKI database vary in size and economic context, suggesting that our approach, which utilizes basic yet widely available data such as demographics and common laboratory tests, might be applicable more broadly.

The focus of this study was on standardizing patient data from diverse hospitals, particularly where non‐electronic records are prevalent, akin to the Chinese context. We developed an AKI prediction model using less than 100 features to maintain both simplicity and functionality as compared to other AKI models required thousands of features, which might not be available in LMICs.^13^, ^28^

Despite international differences in healthcare systems affecting EMR variability, our machine learning model offers a viable framework for developing AKI prediction models, particularly in settings without digital records. We have enhanced the model's interpretability with quantitative analysis and feature importance assessments, contributing valuable insights for multicentric studies across countries.

The proposed predictive model and data processing techniques are designed for versatility, suitable for both non‐standard and standard EMRs, and scalable across a broad spectrum of feature sets. Data preprocessing may be required to tailor the model to specific datasets. In practice, even within the same region or country, data heterogeneity can impact model performance, necessitating adaptive model selection and parameter tuning for optimal results.

Although the performance of machine learning models is contingent on data quality, posing challenges in optimization and generalization, these hurdles do not detract from the utility of machine learning in clinical prediction. Our study highlights the potential and adaptability of our methods and findings.

Fourth, there are concerns about the misclassification of AKI in patients lacking baseline SCr data at admission, a common issue in current research due to the absence of preadmission SCr values. For instance, at Vanderbilt University Hospital in the United States, 57.2% of hospitalized patients lacked preadmission SCr measurements.^29^

To circumvent missing data, the European Renal Best Practice guideline recommends using the initial SCr reading at admission as a proxy baseline if no prior AKI is assumed. Alternatively, the KDIGO guidelines suggest estimating SCr based on a presumed glomerular filtration rate of 75 mL/min/1.73 m^2^, which may overlook undiagnosed chronic kidney disease.^30^

The extent to which missing preadmission SCr affects AKI classification remains under‐investigated, with variations in detection rates from negligible to 24% across studies, depending on the baseline availability.^30^ This challenge is acute in China, where hospital EMR systems do not reliably transfer outpatient SCr data, impeding accurate AKI classification.

Addressing AKI diagnosis at admission requires the evaluation of additional clinical indicators, including urine output changes, symptomatic evidence, previous renal function records, and patient interviews. Nevertheless, the global clinical community faces difficulties with pre‐hospitalization SCr data, exacerbated by the unfeasibility of daily SCr testing before admission. This highlights an ongoing need for enhanced diagnostic methodologies for AKI, prompting a more thorough and systematic approach to fill this critical data gap.

## CONFLICT OF INTEREST STATEMENT

The authors have no conflicts to disclose.

## DATA AVALIABILITY STATEMENT

The datasets analyzed in this study will be available from the corresponding author (Xinling Liang, email: xinlingliang_ggh@163.com) at the time of publication. Per institutional policy, the datasets are designated limited access. Upon receiving access, the investigator may only use them for the purposes outlined in the request to the data provider, and redistribution of the data is prohibited. A demo program of this study was provided at https://www.weoya.com/paper/index?changelanguage=1. Source code for the machine learning model is available at: https://github.com/zaulong/Multi‐center_AKI_Prediction.

## Supporting information



Supporting Information
