# Supplementary material for: Developing machine learning‐driven acute kidney injury predictive models using non‐standard EMRs in resource‐limited settings
Source: Med Phys. 2025 Sep 29;52(10):e70038. doi: 10.1002/mp.70038 (PMC12480061; doi:10.1002/mp.70038)
Supplement: Supplementary file 1 — Supporting Information [file MP-52-0-s001.pdf]

Supplement Materials

Supplement Table 1. Medication category conversion.

Medication Category	Medications
Antibiotics	Aminoglycosides, amino glycopeptides, generation 2 dotomycin, antifungal agent, injection of antifungal agent, ribavirin, penicillin, penicillin+clavulanic acid, cephalosporins, cefoperazone sodium and tazobactam sodium, β-lactamase, miscellaneous antibiotics, sulfanilamide, 4-Quinolones, carbapenem
Diuretic	Diuretics or dehydrant, mannitol, glycerin fructose, fructose, spironolactone, hydrochlorothiazide, furosemide tablets, furosemide injection, furosemide injection, dextran
Hemostatic, nonsteroidal anti-inflammatory drugs (NSAIDs)	NSAIDs
Calcineurin inhibitor (CNI)	Cyclosporine, tacrolimus
Chemotherapy	Alkylating agent, antitumor antiobiotics, antimetabolites, plant alkaloids, others
Iodinated contrast medium	Iodinated contrast agents, ion-hyperosmotic, non-ionic hypotonic monomer, non-ionic isotonic dimer
Renin angiotensin system inhibitors (RASI)	ACEI, ARB, ARB+CCB, ACEI/ARB
Vasoactive	Dopamine, ephedrine, noradrenaline, phenylephrine, epinephrine, isoprenaline
Hemostatic	antifibrinolytic hemostatic, adrenobazonum, etamsylate
Hypophysin	Hypophysin

ACEI: angiotensin-converting enzyme inhibitor; ARB: angiotensin receptor blocker; CCB: calcium channel blocker.

Supplement Table 2. Weights assigned to the different comorbid conditions used to calculate the Charlson total score.

Item	Comorbid condition	Weight
1	Myocardial infarction	1
2	Congestive heart failure	1
3	Peripheral vascular disease	1
4	Cerebrovascular disease	1
5	Dementia	1
6	Chronic pulmonary disease	1
7	Connective tissue disease	1
8	Ulcer disease	1
9	Mild liver disease	1
10	Diabetes	1
11	Hemiplegia	2
12*	Moderate or severe renal disease	2
13	Diabetes with end-organ damage	2
14	Any tumor	2
15	Leukemia	2
16	Lymphoma	2
17	Moderate or severe liver disease	3
18	Metastatic solid tumor	6
19	AIDS	6

* The moderate-to-severe renal disease score was not included in the calculation of the Charlson total score as the patients with moderate-to-severe renal disease have been excluded from this study.

Supplement Table 3. Features created from the clinical variables and related statistical values and augmentations.

Feature Category	# of Features	Clinical Variables	Related Statistical Values and Augmentations
Demographics	2	Age, gender	
In-hospital information	3	Hospital grade, admission department, data collection window*	
Laboratory tests	56	The first, the last blood K value, and the number of blood K tests;<br>the first, the last blood Na value, and the number of blood Na tests;<br>the first, the last blood Cl value, and the number of blood Cl tests;<br>the first, the last blood ALB value, and the number of ALB tests;<br>the first, the last UA value, and the number of UA tests;<br>the first, the last HCT value, and the number of HCT tests;<br>the first, the last CO2CP value, and the number of CO2CP tests;<br>the first, the last HGB value, and the number of HGB tests;	The minimum, maximum, mean and standard deviation values of blood K tests;<br>the minimum, maximum, mean and standard deviation values of blood Na tests;<br>the minimum, maximum, mean and standard deviation values of blood ALB tests;<br>the minimum, maximum, mean and standard deviation values of UA tests;<br>the minimum, maximum, mean and standard deviation values of HCT tests;<br>the minimum, maximum, mean and standard deviation values of CO2CP tests;<br>the minimum, maximum, mean and standard deviation values of HGB tests;
Serum creatinine laboratory tests	7	The first, the last SCr values, and the number of SCr tests	The minimum, maximum, mean and standard deviation values of SCr tests;
Charlson comorbidity index	18	Myocardial infarct, congestive heart failure, peripheral vascular disease, cerebrovascular disease, dementia, chronic pulmonary disease, connective tissue disease, ulcer disease, mild liver disease, diabetes, hemiplegia, diabetes with end organ damage, tumor, leukemia, lymphoma, moderate or severe liver disease, metastasis solid tumor, AIDS.	Charlson total score
Medicatio	21	The number of potential nephrotoxic drugs	

ns		used, days of use of anti-infective drugs, days of use of diuretic drugs, days of use of chemotherapy drugs, days of use of vasoactive drugs, days of use of RASI, days of use of iodinated contrast medium drugs, days of use of hemostatic drugs, days of use of NSAIDs, days of use of CNI; days of use of hypophysin<br>The following are binary value variables: (Yes = 1; No = 0)<br>Use of anti-infective drugs? Use of diuretic drugs? Use of chemotherapy drugs? Use of vasoactive drugs? Use of RASI? Use of iodinated contrast medium drugs? Use of hemostatic drugs? Use of NSAIDs? Use of CNI? Use of hypophysin?
	Grand Total: 107	

* For the AKI patients, data collect window = the AKI testing date - admission date; for non-AKI patients, data collect window = the discharge date - admission date.

ALB: albumin; Cl: chloride; CNI: calcineurin inhibitor; CO_2CP: carbon dioxide combining power; HCT: hematocrit; HGB: hemoglobin; K: potassium; Na: sodium; NSAID: nonsteroidal anti-inflammatory drug; RASI: statins and Renin Angiotensin System Inhibitors; SCr: serum creatinine; UA: blood uric acid.

Supplementary Methods: Cohort Division Strategy

In machine learning modeling process, data can be partitioned into a training set and a test set either randomly or in a targeted manner.[1] Random division entails allocating data to these sets without consideration of the originating hospital. Conversely, in multi-center studies where data are independently collected from various hospitals, a targeted approach involves using hospitals as the basis for data segregation. In targeted approach, certain hospitals' data are designated for model development, while others are reserved for external validation. Our study adopts this targeted method of data allocation. This method is deemed more robust for model performance evaluation compared to random partitioning.

As per Transparent Reporting of a multivariable prediction model for Individual Prognosis or Diagnosis (TRIPOD) guidelines.[1] It effectively accommodates non-random variations between datasets[2] and has gained prominence in recent machine learning-based modeling endeavors.[3,4] In addition, targeted partitioning can potentially achieve better accuracy through targeted modeling. When data is divided into different parts based on certain characteristics (e.g., hospital levels and geographical regions used in this study), partitioned models allow the creation of an ensemble model for each data partition, which can lead to improved predictions, especially when relevant predictors for a target vary across different subgroups of the data.[3]

Our partitioning strategy involved segregating the patients from the 15 hospitals into a training cohort and an independent test cohort based on hospital levels and geographical regions. This was done with the intention of creating a diverse and representative distribution of data, ensuring that both the training and testing datasets encapsulate the variability inherent in different hospital settings and locations.

The partitioning method used in this study capitalizes on the similarity within data from the same hospital while simultaneously acknowledging the diversity across different hospitals. This approach not only reflects variations between centers in a

multi-center study, enhancing the model's generalizability across various settings, but also aligns closely with real-world application scenarios, thereby facilitating model deployment.[1,5]

Supplement Table 4. Fifteen hospitals and the partition.

Hospital	China Hospital Level*	Geographical Region	2016 GDP per Capita（US Dollar）	Included Case Number	Partition
Guangdong Provincial People's Hospital	Tertiary	South	20,873	120051	Training set
Chongzuo People's Hospital	Tertiary	South	5,465	15660	Training set
The Ninth People's Hospital of Chongqing	Tertiary	Southwest	8,515	13782	Training set
Zhejiang Provincial People's Hospital	Tertiary	East	17,852	46068	Training set
The Second Hospital of Anhui Medical University	Tertiary	East	11,785	41999	Training set
The Second Affiliated Hospital of Jilin University	Tertiary	North	11,569	78987	Training set
Dongguan People's Hospital	Tertiary	South	12,159	36830	Training set
Huhhot First Hospital	Tertiary	North	15,182	6940	Training set
Shanghai Ninth People's Hospital	Tertiary	East	17,141	22445	Training set
The First People's Hospital of Kashgar	Secondary	Northwest	2,479	48906	Training set
Guangdong Lufeng People's Hospital	Secondary	South	2,723	13471	Training set
Wuhua People's Hospital	Secondary	South	1,903	7708	Test set
Sichuan Provincial People's Hospital	Tertiary	Southwest	11,318	84053	Test set
Xinjiang Uygur Autonomous Region People's Hospital	Tertiary	Northwest	10,230	5296	Test set
Inner Mongolia Autonomous Region People's Hospital	Tertiary	North	15,182	18941	Test set

* The 3-tier Classification of Chinese hospitals is according to the Ministry of Health of the People's Republic of China, which is based on a hospital's ability to provide medical care, medical education, and conduct medical research [6].

Supplement Table 5. Demographic and clinical information of the patients (n=561,137).

Demographics and clinical features	AKI (n=45,610)	non-AKI (n=515,527)	P-value
Age (SD), y	58.008 (17.1)	56.054 (16.8)	<0.05
Sex, n (%)			
Female	20,031 (43.9)	206,827 (40.1)	<0.05
Male	25,579 (56.1)	308,700 (59.9)	
Admission department, n (%)			
Traditional Chinese Medical Science	157 (0.3)	2,316 (0.4)	<0.05
General Family Medicine	452 (1.0)	6,040 (1.2)	
Medical	14,850 (32.6)	186,337 (36.1)	
Oral Cavity	461 (1.0)	10,630 (2.1)	
Surgery	17,121 (37.5)	172,243 (33.4)	
Obstetrics And Gynecology Department	2,156 (4.7)	32,178 (6.2)	
Rehabilitation Medicine and Physical Therapy	90 (0.197)	1,122 (0.218)	
Intensive Care Unit	5,770 (12.7)	32,505 (6.3)	
Dermatology And Venereology	84 (0.2)	3,452 (0.7)	
Ophthalmology	71 (0.2)	1,727 (0.3)	
Neurology	1,835 (4.0)	23,317 (4.5)	
Psychiatry and Mental Health	209 (0.5)	2,361 (0.5)	
Geratology	507 (1.1)	6,747 (1.3)	
Otolaryngology	182 (0.4)	3,945 (0.8)	
Oncology	1,656 (3.6)	30,607 (5.9)	
Hospital grade, n (%)			
Tertiary	42,506 (93.2)	481,792 (93.5)	<0.05
Secondary	3,104 (6.6)	52,090(6.5)	
Data collection window (SD), d	7.311 (5.348)	12.828 (6.506)	<0.05
Charlson total score	2.159 (2.532)	1.774 (2.256)	<0.05
First K (SD), mmol/L	3.880 (0.588)	3.921 (0.534)	<0.05
Last K (SD), mmol/L	3.918 (0.595)	3.940 (0.463)	<0.05
First Na (SD), mmol/L	139.036 (28.672)	139.619 (22.257)	<0.05
Last Na (SD), mmol/L	141.950 (74.988)	139.489 (23.340)	<0.05
First CL (SD), mmol/L	102.640 (6.118)	103.015 (4.944)	<0.05

Last CL (SD), mmol/L	102.640 (6.666)	103.010 (4.629)	<0.05
First ALB (SD), g/L	36.198 (7.130)	38.107 (6.401)	<0.05
Last ALB (SD), g/L	35.355 (7.723)	37.268 (7.544)	<0.05
First UA (SD), μmol/L	328.966 (142.883)	328.951 (115.667)	<0.05
Last UA (SD), μmol/L	295.172 (145.883)	279.498 (116.497)	<0.05
First HCT (SD), %	37.567 (7.473)	38.705 (6.686)	<0.05
Last HCT (SD), %	35.318 (7.349)	36.498 (6.390)	<0.05
First CO2CP (SD), mmHg	24.944 (3.656)	25.024 (3.393)	<0.05
Last CO2CP (SD), mmHg	24.895 (3.789)	25.287 (3.383)	<0.05
First HGB (SD), g/L	124.264 (26.960)	128.208 (23.921)	<0.05
Last HGB (SD), g/L	117.618 (26.634)	121.044 (22.784)	<0.05
First SCr (SD), μmol/L	76.877 (59.190)	77.370 (30.549)	<0.05
Last SCr (SD), μmol/L	71.280 (50.800)	71.179 (18.390)	<0.05
Days of Use of Anti-Infective Drugs (SD), n	4.565 (6.497)	5.764 (8.180)	0.485
Days of Use of Diuretic Drugs (SD), n	3.060 (5.334)	2.426 (6.037)	<0.05
Days of Use of Vasoactive Drugs (SD), n	0.725 (2.478)	0.230 (1.823)	<0.05
Days of Use of RASI (SD), n	0.914 (2.740)	1.625 (4.316)	<0.05
Days of Use of iodinated contrast medium drugs (SD), n	0.021 (0.174)	0.028(0.235)	<0.05
Days of Use of Hemostatic Drugs (SD), n	0.466 (2.219)	0.437 (2.163)	<0.05
Days of Use of Chemotherapy Drugs (SD), n	2.382 (5.626)	4.644 (8.266)	<0.05
Days of Use of NSAIDs (SD), n	0.384 (1.494)	0.635 (2.349)	<0.05
Days of Use of CNI (SD), n	0.024 (0.605)	0.030 (0.737)	0.108
Days of Use of Hypophysin (SD), n	0.025 (0.482)	0.018 (0.412)	<0.05
Use of anti-infective drugs, n (%)	26,276 (57.6)	236,192 (45.8)	<0.05
Use of diuretic drugs, n (%)	17,936 (39.3)	113,539 (22.0)	<0.05
Use of vasoactive drugs, n (%)	7,072 (15.5)	18,059(3.5)	<0.05
Use of RASI drugs, n (%)	6,552 (14.4)	81,528 (15.8)	<0.05

Use of iodinated contrast medium drugs, n (%)	617 (1.3)	8,103 (1.6)	<0.05
Use of hemostatic drugs, n (%)	3,466 (7.6)	31,861 (6.2)	<0.05
Use of chemotherapy drugs, n (%)	11,545 (25.3)	170,811 (33.1)	<0.05
Use of NSAIDs drugs, n (%)	4,804 (10.5)	58,267 (11.3)	<0.05
Use of CNI drugs, n (%)	107 (0.2)	1,010 (0.2)	0.118
Use of hypophysin drugs, n (%)	215 (0.5)	1,332 (0.3)	<0.05

AKI: acute kidney injury; K: potassium; Na: sodium; Cl: chloride; ALB: albumin; UA: blood uric acid; HCT: hematocrit; CO_2CP: carbon dioxide combining power; HGB: hemoglobin; SCr: serum creatinine; RASI: statins and Renin Angiotensin System Inhibitors; NSAID: nonsteroidal anti-inflammatory drug; CNI: calcineurin inhibitor.

Supplement Table 6. Comparisons of features between the training cohort and the testing cohort.

Demographics and clinical characteristics	Training cohort (n=445139)	Test cohort (n=115998)	P-value
Age (SD), y	55.919 (16.746)	57.343 (17.325)	<0.05
Sex, n (%)			<0.05
Female	183,385 (41.197)	43,473 (37.477)	
Male	261,754 (58.803)	72,525 (62.523)	
Admission department, n (%)			<0.05
Traditional Chinese Medical Science	2,095 (0.471)	378 (0.326)	
General Family Medicine	5,687 (1.278)	805 (0.694)	
Medical	163,332 (36.692)	37,855 (32.634)	
Oral Cavity	10,946 (2.459)	2,219 (1.911)	
Surgery	147,479 (33.131)	41,885 (36.108)	
Obstetrics And Gynecology Department	28,759 (6.461)	5,584 (4.814)	
Rehabilitation Medicine and Physical Therapy	976 (0.219)	236(0.203)	
Intensive Care Unit	27,478 (6.173)	10,797 (9.308)	
Dermatology And Venereology	1,141 (0.256)	2,395 (2.065)	
Ophthalmology	1,648 (0.370)	754 (0.650)	
Neurology	19,854 (4.460)	5,298 (4.567)	
Psychiatry and Mental Health	2,287 (0.514)	236 (0.203)	
Geratology	1,968 (0.442)	5,286 (4.557)	
Otolaryngology	3,373 (0.758)	754 (0.650)	
Oncology	28,116 (6.316)	4,147 (3.575)	
Hospital grade, n (%)			0.221
Tertiary	416,008 (93.456)	108,290 (93.355)	
Secondary	29,131 (6.544)	7,708 (6.645)	
Data collection window (SD), d	12.055 (6.538)	13.626 (6.662)	<0.05
Charlson total score	1.735 (2.149)	2.033 (2.651)	<0.05
First K (SD), mmol/L	3.912 (0.507)	3.939 (0.642)	<0.05
Last K (SD), mmol/L	3.932 (0.477)	3.964 (0.467)	<0.05
First Na (SD), mmol/L	139.675 (25.625)	139.197 (4.578)	<0.05
Last Na (SD), mmol/L	139.780 (34.652)	139.332 (34.034)	<0.05
First CL (SD), mmol/L	102.761 (5.110)	103.607 (4.780)	<0.05
Last CL (SD), mmol/L	102.829 (4.743)	103.607 (4.780)	<0.05
First ALB (SD), g/L	37.815 (6.476)	38.516 (6.446)	<0.05
Last ALB (SD), g/L	37.355 (7.952)	36.325 (5.963)	<0.05
First UA (SD), μmol/L	328.779 (118.180)	329.585 (116.798)	<0.05
Last UA (SD), μmol/L	281.807 (120.655)	276.530 (112.758)	<0.05
First HCT (SD), %	38.552 (6.751)	41.208 (6.541)	<0.05

Last HCT (SD), %	36.489 (6.513)	39.931 (6.484)	<0.05
First CO2CP (SD), mmHg	25.079 (3.342)	24.291 (4.114)	<0.05
Last CO2CP (SD), mmHg	25.285 (3.374)	24.905 (3.899)	<0.05
First HGB (SD), g/L	127.639 (23.828)	128.918 (25.583)	<0.05
Last HGB (SD), g/L	120.336 (22.954)	122.506 (23.732)	<0.05
First SCr (SD), μmol/L	76.273 (33.939)	81.275 (32.720)	<0.05
Last SCr (SD), μmol/L	70.241 (23.088)	74.714 (21.069)	<0.05
Days of Use of anti-infective drugs (SD), n	5.473 (7.967)	6.308 (8.338)	<0.05
Days of Use of diuretic drugs (SD), n	2.567 (6.047)	2.181 (5.761)	<0.05
Days of Use of vasoactive drugs (SD), n	0.279 (1.980)	0.243 (1.554)	<0.05
Days of Use of RASI (SD), n	1.642 (4.186)	1.312 (4.299)	<0.05
Days of Use of iodinated contrast medium drugs (SD), n	0.030 (0.233)	0.010 (0.145)	<0.05
Days of Use of Hemostatic Drugs (SD), n	0.356 (2.008)	0.718 (2.614)	<0.05
Days of Use of Chemotherapy Drugs (SD), n	3.957 (7.347)	6.132 (10.061)	<0.05
Days of Use of NSAIDs (SD), n	0.670 (2.307)	0.431 (2.232)	<0.05
Days of Use of CNI (SD), n	0.020 (0.579)	0.062 (1.083)	<0.05
Days of Use of Hypophysin (SD), n	0.011 (0.302)	0.043 (0.674)	<0.05
Use of anti-infective drugs, n (%)	197,025 (44.261)	65,443 (56.417)	<0.05
Use of diuretic drugs, n (%)	108,245 (24.317)	23,230 (20.026)	<0.05
Use of vasoactive drugs, n (%)	20,030 (4.500)	5,101 (4.397)	<0.05
Use of RASI drugs, n (%)	73,461 (16.503)	14,619 (12.603)	<0.05
Use of iodinated contrast medium drugs, n (%)	7,871 (1.768)	849 (0.732)	<0.05
Use of hemostatic drugs, n (%)	21,789 (4.895)	13,538 (11.671)	<0.05
Use of chemotherapy drugs, n (%)	130,237 (29.258)	52,119 (44.931)	<0.05
Use of NSAIDs drugs, n (%)	56,306 (12.649)	6,765 (5.832)	<0.05
Use of CNI drugs, n (%)	614 (0.138)	503 (0.434)	<0.05
Use of hypophysin drugs, n (%)	960 (0.216)	587 (0.506)	<0.05

K: potassium; Na: sodium; Cl: chloride; ALB: albumin; UA: blood uric acid; HCT: hematocrit; CO2CP: carbon dioxide combining power; HGB: hemoglobin; SCr: serum creatinine; RASI: statins and Renin Angiotensin System Inhibitors; NSAID: nonsteroidal anti-inflammatory drug; CNI: calcineurin inhibitor.

Supplement Table 7. Top features of different prediction models.

Features	24h		48h		72h	
	SCr	w/o SCr	SCr	w/o SCr	SCr	w/o SCr
Data collection window	√	√	√	√	√	√
hospital department	√	√	√	√	√	√
days of use of anti-infective drugs	√	√	√	√	√	√
age	√	√	√	√	√	√
last CL	√	√	√	√	√	√
standard deviation UA	√	√	√	√		√
the number of potential nephrotoxic drugs used	√	√	√	√	√	√
last Na	√	√	√	√	√	√
last UA	√	√	√	√	√	√
days of use of diuretic drugs	√	√	√	√	√	√
last K	√	√	√	√		
days of use of chemotherapy drugs	√	√	√	√	√	√
UA test times		√		√		√
first ALB	√	√	√	√	√	√
standard deviation HGB	√	√				√
minimum ALB	√	√	√	√		√
last CO2CP	√	√	√	√		√
minimum HGB		√		√	√	√
first Na		√				
standard deviation CL		√		√		
minimum UA		√				
last ALB		√	√	√	√	
CO2CP test times		√		√		√
standard deviation K		√				
HCT test times	√	√		√		
standard deviation Na		√				
first K		√				
minimum HCT	√	√	√		√	√
K test times		√				√
standard deviation ALB		√		√		√
standard deviation SCr	√		√		√	
last SCr	√		√		√	
maximum SCr	√		√		√	
minimum SCr	√		√		√	
first CO2CP	√		√	√	√	√
first SCr	√		√		√	
last HGB	√					
first HCT	√					

Charlson total score	√	√	√	√	√
standard deviation HCT	√				
days of use of vasoactive drugs	√			√	√
SCr test times	√	√		√	
minimum CL		√	√	√	
Congestive heart failure		√		√	√
first CL		√	√		
last HCT		√		√	
first UA			√		√
first HGB			√	√	√
minimum CO2CP			√		√
mean K			√		
HGB test times				√	
hospital grade				√	
Myocardial infarction				√	√
CL test times					√

Check mark denotes the feature occurs in top lists.

w/o SCr: without serum creatinine; Cl: chloride; UA: blood uric acid; Na: sodium; K: potassium; ALB: albumin; HGB: hemoglobin; ALB: albumin; CO_2CP: carbon dioxide combining power; HCT: hematocrit; SCr: serum creatinine.

References

1. Collins GS, Reitsma JB, Altman DG, Moons KG. Transparent reporting of a multivariable prediction model for individual prognosis or diagnosis (TRIPOD): the TRIPOD statement. *BMJ*. Jan 7 2015;350:g7594. doi:10.1136/bmj.g7594
10.1136/bmj.g7594.
2. Mehta RL, Cerda J, Burdmann EA, et al. International Society of Nephrology's 0by25 initiative for acute kidney injury (zero preventable deaths by 2025): a human rights case for nephrology. *Lancet*. Jun 27 2015;385(9987):2616-43. doi:10.1016/S0140-6736(15)60126-X
3. Pieszko K, Hiczkiewicz J, Lojewska K, et al. Artificial intelligence in detecting left atrial appendage thrombus by transthoracic echocardiography and clinical features: the Left Atrial Thrombus on Transoesophageal Echocardiography (LATTEE) registry. *Eur Heart J*. Jan 1 2024;45(1):32-41. doi:10.1093/eurheartj/ehad431
4. D'Ascenzo F, De Filippo O, Gallone G, et al. Machine learning-based prediction of adverse events following an acute coronary syndrome (PRAISE): a modelling study of pooled datasets. *Lancet*. Jan 16 2021;397(10270):199-207. doi:10.1016/S0140-6736(20)32519-8
5. Alosco ML, Barr WB, Banks SJ, et al. Neuropsychological test performance of former American football players. *Alzheimers Res Ther*. Jan 3 2023;15(1):1. doi:10.1186/s13195-022-01147-9
10.1186/s13195-022-01147-9.
6. Li L, Du T, Zeng S. The Different Classification of Hospitals Impact on Medical Outcomes of Patients in China. *Front Public Health*. 2022;10:855323. doi:10.3389/fpubh.2022.855323
